# Characterization of drug resistance mutations in people living with HIV with low-level viremia on antiretroviral therapy in Chongqing, China

**DOI:** 10.3389/fpubh.2026.1753298

**Published:** 2026-02-06

**Authors:** Huizheng Zhang, Zhen Zhang, Wei Ye, Weidong Pan

**Affiliations:** 1 Department of Clinical Laboratory, Chongqing Public Health Medical Center, Chongqing, China; 2 Department of Pharmacy, Chongqing Public Health Medical Center, Chongqing, China; 3 Department of Physical Medicine and Rehabilitation, Chongqing General Hospital, Chongqing University, Chongqing, China; 4 Department of Immunology, College of Basic Medical Sciences, Zhengzhou University, Zhengzhou, Henan, China

**Keywords:** antiretroviral therapy, Chongqing, drug resistance mutations, genotyping, human immunodeficiency virus type 1, low-level viremia, RNA/DNA

## Abstract

**Objective:**

To investigate the prevalence and patterns of drug resistance mutations (DRMs) and associated risk factors among ART-experienced people living with HIV (PLWH) exhibiting low-level viremia (LLV; 50–999 copies/mL) in Chongqing, China, and to evaluate the utility of a combined plasma RNA and proviral DNA genotyping strategy.

**Methods:**

In this cross-sectional study (September 2023–February 2024), we screened 4,941 ART-treated individuals, identifying 210 with LLV. Genotypic resistance testing targeting the protease, reverse transcriptase, and integrase regions was successfully performed for 133 participants (63.33%) using a dual-source (plasma RNA and/or proviral DNA) approach. HIV-1 subtyping and DRM analysis were conducted using the Stanford HIVdb algorithm and phylogenetic methods.

**Results:**

The overall prevalence of LLV was 4.25%. CRF07_BC was the predominant HIV-1 subtype (76.69%). DRMs were detected in 26.32% (35/133) of the cohort. Resistance to non-nucleoside reverse transcriptase inhibitors (NNRTIs) was most common (19.55%), primarily driven by V179D/E and G190A/S/E mutations. Resistance to nucleoside reverse transcriptase inhibitors (NRTIs) and integrase strand transfer inhibitors (INSTIs) was observed in 12.03 and 3.76% of participants, respectively, with M184V/I being the predominant NRTI mutation. No significant differences in DRM prevalence were observed across viral subtypes. In multivariate analysis, baseline CD4^+^ T-cell count <100 cells/μL was significantly associated with DRMs specifically in the isolated LLV subgroup.

**Conclusion:**

This study reveals a considerable prevalence of drug mutations (DRMs), predominantly against NNRTIs, among ART-experienced PLWH with LLV in Chongqing. The combined RNA/DNA genotyping strategy significantly enhanced DRM detection sensitivity in this low viral load cohort, underscoring its value for resistance surveillance and guiding clinical management in resource-limited settings.

## Background

Low-level viremia (LLV) represents a persistent therapeutic challenge in the management of people living with HIV (PLWH) on antiretroviral therapy (ART). There is no global consensus on its definition: the World Health Organization (WHO) defines LLV as a viral load (VL) between 50 and 1,000 copies/mL (1), whereas U.S. guidelines set a narrower range of 50–200 copies/mL (2), and European guidelines adopt an even lower threshold (3). This definitional ambiguity complicates the clinical management of individuals in this virologic “gray zone,” situated between sustained suppression and overt treatment failure.

The clinical significance of LLV is underscored by its association with an increased risk of subsequent virologic failure (VF) (4–7) and the emergence of drug resistance mutations (DRMs) (5, 7, 8). DRMs during LLV are a critical independent predictor of VF, elevating the importance of effective resistance surveillance. However, conventional genotypic resistance testing, which relies on plasma viral RNA, often requires a VL > 1,000 copies/mL for reliable amplification, leaving a diagnostic gap for a significant portion of the LLV population. International guidelines reflect this dilemma, offering conflicting recommendations on when to test, often driven by cost-effectiveness concerns in resource-limited settings. Proviral DNA genotyping (9), which targets the integrated viral reservoir in host cells, presents a viable complementary strategy to overcome the limitations of low plasma viremia, enabling the detection of archived resistance.

Geographically, the prevalence of LLV and its associated DRM burden vary considerably, influenced by local ART regimens, treatment histories, and monitoring practices. In China, reported LLV rates range from 10 to 30% (10, 11), with DRM rates in LLV cohorts varying from 42.3% in Henan (11) to 47.06% in Guangdong (12). Of particular note is Chongqing, a high-prevalence region in southwestern China with a large and rapidly growing population of people living with HIV (approximately 72,000 surviving cases as of October 2023, ranking sixth nationally) (13), faces a pressing need for local epidemiological data. The region’s distinct ART rollout history, characterized by extensive prior use of non-nucleoside reverse transcriptase inhibitor (NNRTI)-based regimens, likely shapes a unique resistance landscape. A persistent lack of comprehensive data on LLV and DRMs in Chongqing has hindered the development of tailored clinical strategies for this population.

Therefore, this cross-sectional study aimed to fill this critical knowledge gap by systematically investigating the prevalence and patterns of DRMs among ART-experienced PLWH with LLV in Chongqing. We employed a combined plasma RNA and proviral DNA genotyping approach to enhance detection sensitivity. Our objectives were threefold: to elucidate the local DRM profile, to evaluate the utility of the integrated genotyping strategy in a low-VL cohort, and to identify risk factors associated with DRMs, thereby providing an evidence base to optimize clinical management and inform national policy.

## Materials and methods

### Study population

This cross-sectional observational study was conducted at the Chongqing Public Health Medical Center, a large tertiary specialized infectious disease hospital in Southwest China. At the time of the study, the center served a large cohort of PLWH. An estimated approximately 10,000 individuals regularly monitored for plasma VL and CD4^+^ T-cell counts was derived from the hospital’s laboratory information system, based on the annual average number of unique patients undergoing these tests in recent years (e.g., 2023 and 2024). Due to funding constraints and prolonged research approval processes, this study collected information from patients who underwent pVL testing at the center between September 2023 and February 2024. Individuals meeting all the following criteria were included in the analysis: (1) Received ART for at least 24 weeks; (2) Maintained sustained virologic suppression during treatment, defined as all available VL results being <50 copies/mL; (3) Subsequently experienced virologic breakthrough, meeting one of the following two patterns: Isolated Low-Level Viremia or Blips (iLLV/Blips) that was defined as aa single VL measurement >50 copies/mL but <1,000 copies/mL among multiple (≥3) VL tests, with all other time-point VL results being ≤50 copies/mL or ≥1,000 copies/mL. Persistent Low-Level Viremia (pLLV) that was defined as consecutive (≥2) VL measurements >50 copies/mL but <1,000 copies/mL among multiple (≥3) VL tests.

Remaining plasma and whole blood samples routinely stored after VL testing during an LLV episode were retrieved. Demographic data and medical records, including HIV-VL, CD4^+^ T-cell counts, and transmission routes, were collected. The specific variables presented in this study (e.g., demographic characteristics, baseline and current CD4^+^ counts, VL values, transmission route, time from diagnosis to ART initiation, and initial treatment regimen) were extracted from the hospital’s electronic medical record system. The definitions of these variables followed standard clinical and laboratory protocols: dates of diagnosis and ART initiation were obtained from medical records; transmission route was based on self-reported information documented at enrollment; CD4^+^ counts and VL were measured in our laboratory using standardized assays. Although this study utilized stored samples and retrospective data, these variables were derived from real-time clinical and laboratory results recorded during routine HIV management, thereby ensuring their validity for cross-sectional analysis.

### Nucleic acid extraction

For proviral DNA analysis, we utilized archived whole blood samples stored at −80 °C. Genomic DNA was isolated from these samples employing a commercial DNA extraction kit (Shuoshi, Jiangsu, China) in strict adherence to the manufacturer’s protocol, and the resulting DNA was stored for downstream applications.

For viral RNA extraction, cryopreserved (−80 °C) residual plasma aliquots (0.5 mL) were processed. To enhance detection sensitivity, viral particles were first concentrated from plasma using an exosome enrichment reagent (Hailite, Guangzhou). Subsequently, viral RNA was purified from the concentrate using a specialized RNA isolation kit (Hailite, Guangzhou), as per the provided guidelines.

### Genotypic drug resistance testing

Validated in-house methods (14) were used to amplify the target gene fragments: the protease and reverse transcriptase regions of the HIV-1 pol gene (~1,500 bp) and the integrase (IN) gene region (~1,200 bp). Specimens with insufficient one-step RT-PCR amplification products underwent nested PCR. A GeneAmp^®^ 9700 PCR system (Applied Biosystems, USA) was used for both rounds of amplification. Target bands were verified by 1% agarose gel electrophoresis, and the amplification products were sequenced. Sequences were assembled, edited, and submitted to the Stanford University HIV Drug Resistance Database^1^ for HIV-1 subtyping and DRM analysis. The PCR primer list is provided in Table 1. Polymorphic mutations and accessory mutations were included in our analysis as they, in combination with other DRMs, can lead to reduced susceptibility to certain antiretroviral drugs.

**Table 1:** Primers for amplification of HIV-1 *pol* genes.

Procedure	Primers	Position^a^	Length of target fragment	Sequences (5′-3′)
First round	2029F	2029–2050	1,501	TGGAAATGTGGRAAGGAAGGAC
	3529R	3,529–3,505		GCTAyyAAGTCTTTTGATGGGTCAT
	4007F	4,007–4,030	1,213	GCAGGATTCRGGATYAGAAGTAAA
	5219R	5,219–5,243		CCTAGTGGGATGTGTACTTCTGAAC
Second round	2249F	2,249–2,266	1,273	CTTCCCTCARATCACTCT
	3521R	3,521–3,504		GTCTTTTGATGGGTCATA
	4063F	4,063–4,080	1,157	TCATTCARGCACAACCAG
	5219 R	5,219–5,243		CCTAGTGGGATGTGTACTTCTGAAC

a Nucleotide positions with reference to HIV HXB2 strain (GenBank accession number: K03455).

### Subtyping and phylogenetic analysis

For subtype analysis, the REGA HIV-1 Subtyping Tool version 3.0^2^ was used to determine the subtype of HIV-1 isolates based on partial pol sequences, which was further confirmed by phylogenetic analysis. Phylogenetic analysis was performed using Molecular Evolutionary Genetics Analysis (MEGA) software version XI, employing the maximum likelihood method and the General Time Reversible model. Tree topology was tested by bootstrap analysis with 1,000 replicates. Reference sequences included in the maximum likelihood tree (GenBank Accession Nos. U51189, AF286226, AF286229, AF069670, AY945737, DQ207940, U21135, AF067155, JX574661, AF077336, AF061642, AF190127, AF082395, AJ249235, AF286236) were downloaded from the Los Alamos HIV Sequence Database.^3^

### Statistical analysis

Statistical analyses were performed using IBM SPSS Statistics for Windows, Version 27. Normally distributed continuous variables are presented as mean ± standard deviation, while non-normally distributed variables are presented as median and interquartile range. Categorical variables are presented as numbers and percentages. For analysis, key continuous variables (e.g., baseline CD4^+^ T-cell count, VL during the LLV episode) were categorized using clinically meaningful thresholds. The primary outcome was the presence of any drug resistance mutation (DRM), defined as a binary variable (yes/no) based on the detection of major or accessory mutations listed in the Stanford HIVdb algorithm. Comparisons between groups for categorical variables were performed using the Chi-square test or Fisher’s exact test, as appropriate. Univariate and multivariate logistic regression analyses were employed to identify factors associated with DRMs. A two-sided *p*-value < 0.05 was considered statistically significant.

## Results

### Study cohort and genotyping success

**Figure 1:**
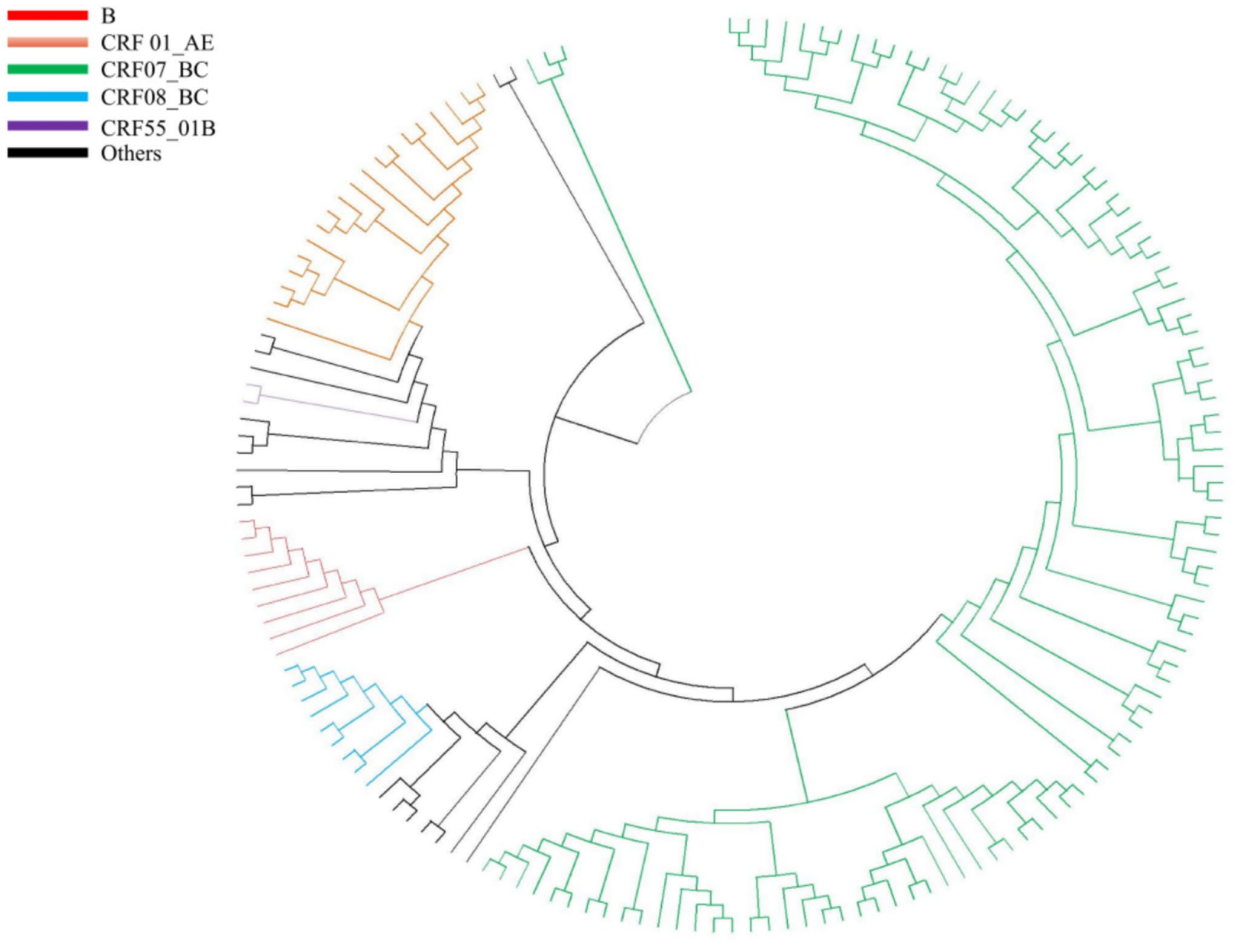
Phylogenetic tree based on *pol* sequence was constructed using Molecular Evolutionary Genetic Analysis (MEGA) software (version 11) based on neighbor-joining method and general time reversible model with 250 bootstrap replicates. Different subtypes are shown in different colors. Reference sequences (GenBank No. U51189, AF286226, AF286229, AF069670, AY945737, DQ207940, U21135, AF067155, JX574661, AF077336, AF061642, AF190127, AF082395, AJ249235, AF286236) were downloaded from the Los Alamos HIV Sequence Database (https://www.hiv.lanl.gov/).

Among 4,941 ART-treated individuals, 210 (4.25%) were identified as having LLV. The genotyping success rate for the pol and integrase genes was 63.33%, achieved in 133 of the 210 individuals with LLV. The final analysis cohort was predominantly male (74.44%, 99/133) and aged 50 years or older (51.13%, 68/133). Heterosexual contact was the leading transmission route (78.95%, 105/133), followed by men who have sex with men (MSM, 18.80%, 25/133). At ART initiation, the median baseline CD4^+^ T-cell count was 171 cells/μL (range: 6–742), and the median baseline VL was 90,000 copies/mL (range: 107–16,200,000). At the time of LLV detection, the median CD4^+^ T-cell count was 317 cells/μL (range: 18–1,067), and the median VL was 192 copies/mL (range: 51–980) (Table 2). HIV RNA sequencing was successful in 70.68% (94/133) of the genotyped samples. The CRF07_BC subtype was overwhelmingly dominant (76.69%, 102/133), with CRF01_AE (12.78%, 17/133) and CRF08_BC (6.77%, 9/133) being the next most prevalent. Subtype C and B were identified in 3.01% (4/133) and 0.75% (1/133) of participants, respectively (Table 3). These subtyping results were robustly confirmed by phylogenetic analysis (Figure 1).

**Table 2:** Univariate logistic regression analysis of factors associated with drug resistance mutations in the overall low-level viremia cohort (*N* = 133).

Variables	All (*N* = 133)		DRMs (*N* = 35)		OR	Lower bound	Upper bound	*p*-value
Sex, *n* (%)	133	100.00%	35	26.32%				
Male	99	74.44%	26	26.26%	0.989	0.409	2.394	0.981
Female	34	25.56%	9	26.47%	1			
Age (years), *n* (%)								
<30	14	10.53%	2	14.29%	0.403	0.078	2.079	0.277
30–39	29	21.80%	10	34.48%	1.272	0.459	3.525	0.644
40–49	22	16.54%	5	22.73%	0.711	0.213	2.367	0.578
50–59	27	20.30%	6	22.22%	0.690	0.223	2.136	0.520
≥60	41	30.83%	12	29.27%	1			
Transmission category, *n* (%)								
MSM	25	18.80%	5	20.00%	1			
HSX	105	78.95%	30	28.57%	1.600	0.550	4.653	0.388
Other	3	2.26%	0	0.00%	0.000	0.000		0.999
WHO classification stage, *n* (%)								
1	66	49.62%	17	25.76%	1			
2	2	1.50%	1	50.00%	2.882	0.171	48.656	0.463
3	19	14.29%	5	26.32%	1.029	0.323	3.286	0.961
4	46	34.59%	12	26.09%	1.017	0.431	2.401	0.969
CD4^+^, median (min/max), cells/μL	171 (6/742)							
Baseline CD4^+^ T-cell count, *n* (%)								
<100	44	33.08%	16	36.36%	2.413	0.932	6.247	0.070
100–250	42	31.58%	10	23.81%	1.319	0.478	3.644	0.593
≥250	47	35.34%	9	19.15%	1			
VL, median (min/max), copies/mL	90,000 (107/16200000)							
Baseline VL, *n* (%)								
<10,000	46	34.59%	17	36.96%	1			
10,000–100,000	22	16.54%	3	13.64%	0.269	0.069	1.046	0.058
100,000–1,000,000	46	34.59%	9	19.57%	0.415	0.162	1.065	0.067
≥1,000,000	19	14.29%	6	31.58%	0.787	0.252	2.456	0.680
CD4^+^, median (min/max), cells/μL	317 (18/1067)							
CD4^+^ count during LLV, *n* (%)								
<200	40	30.08%	11	27.50%	0.786	0.306	2.017	0.616
200–450	50	37.59%	10	20.00%	0.518	0.202	1.328	0.171
≥450	43	32.33%	14	32.56%	1			
VL During LLV, median (min/max), copies/mL	192 (51/980)							
VL During LLV, copies/mL								
<150	49	36.84%	10	20.41%	1			
150–300	40	30.08%	9	22.50%	1.132	0.410	3.129	0.811
≥300	44	33.08%	16	36.36%	2.229	0.882	5.633	0.090
Time from diagnosis to initiation of ART, year								
Unknown	55	41.35%	18	32.73%	1			
<1	39	29.32%	5	12.82%	0.302	0.101	0.903	0.032
1–2	19	14.29%	6	31.58%	0.949	0.310	2.906	0.927
≥2	20	15.04%	6	30.00%	0.881	0.290	2.673	0.823
Initial treatment regimen								
2NRTIs + NNRTIs	81	60.90%	25	30.86%	1			
2NRTIs + PIs	6	4.51%	0	0.00%	0.000	0.000		0.999
2NRTIs + INSTIs	8	6.02%	1	12.50%	0.320	0.037	2.741	0.298
NRTIs + INSTIs	32	24.06%	9	28.13%	0.877	0.355	2.163	0.775
Other	6	4.51%	0	0.00%	0.000	0.000		0.999

MSM, men who have sex with men; HSX, heterosexual contact; iLLV, isolated low-level viremia; pLLV, persistent low-level viremia; NRTI, nucleoside reverse transcriptase inhibitor; NNRTI, non-nucleoside reverse transcriptase inhibitor; INSTI, integrase strand transfer inhibitor. Statistical analysis: univariate logistic regression was used to calculate odds ratios (OR) with 95% confidence intervals (CI). The reference category (Ref) for each variable is indicated.

Variable selection and excluded data: Marital Status, Occupation, and Education Level: These variables were excluded from the final presented analysis because they showed no significant association with DRMs in univariate analysis (all *p*-values > 0.2), and their numerous categories contributed to table complexity without adding explanatory power to the primary research question. Their distributions were as follows: Marital status (Unmarried: 18.8%, Married: 45.1%, Divorced: 12.0%, Widowed: 15.0%, Unknown: 9.0%); Occupation (Unemployed: 35.3%, Farmer: 14.3%, Staff: 11.3%, etc.); Education (Primary or below: 30.8%, Secondary: 34.6%, College or above: 22.6%, Unknown: 12.0%).

**Table 3:** Distribution of drug resistance mutations (DRMs) by HIV-1 subtypes.

Subtype	Patients		DRMs		*χ* ^2^	*p*-value
	*n*	%	*n*	%		
B	1	0.75%	1	100.00%	3.288	0.511
C	4	3.01%	1	25.00%		
CRF01_AE	17	12.78%	5	29.41%		
CRF07_BC	102	76.69%	25	24.51%		
CRF08_BC	9	6.77%	3	33.33%		
Total	133	100.00%	35	26.32%		

### Spectrum of drug resistance mutations

**Figure 2:**
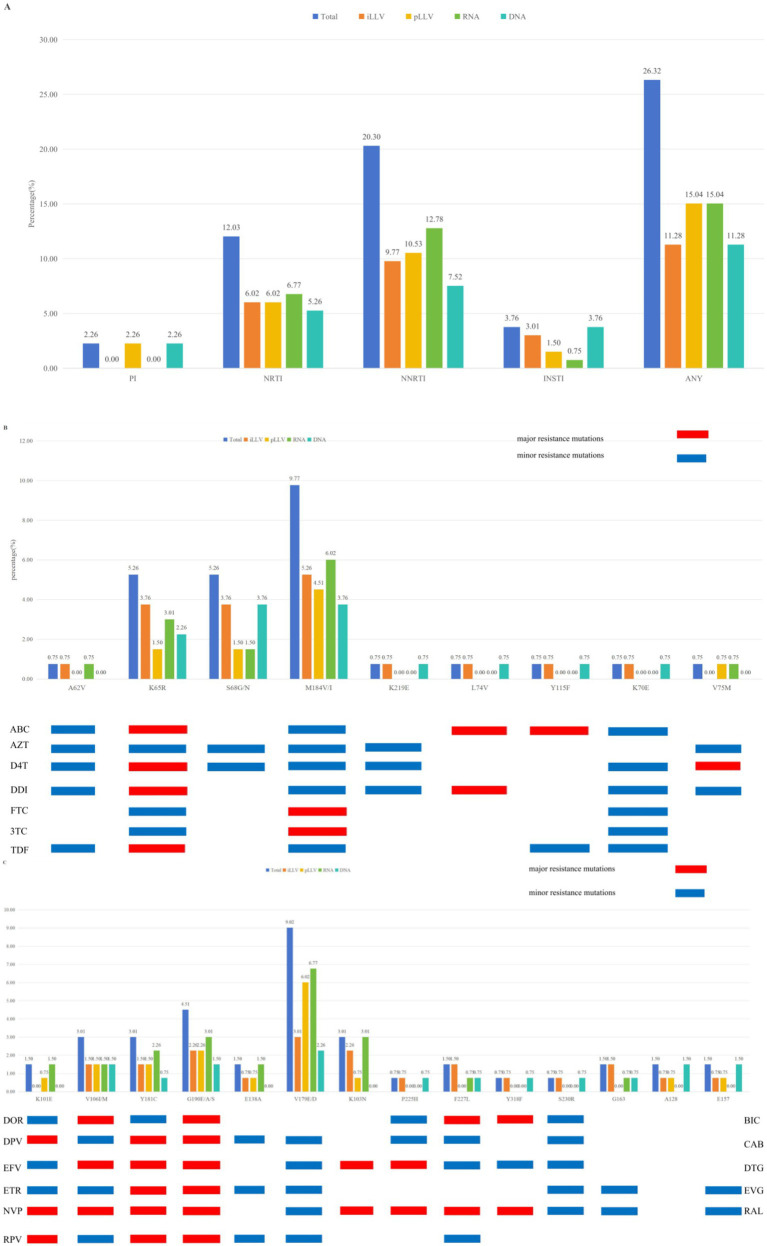
Distribution and prevalence of drug class-specific DRMs in PLWH with LLV in Chongqing. **(A)** Prevalence of DRMs for four drug classes (NNRTI, NRTI, PI, and INSTI); **(B)** specific DRMs stratified by NRTI; **(C)** specific DRMs stratified by NNRTI and INSTI. The major and minor resistance mutations are highlighted in red and blue, respectively. NNRTI, non-nucleoside reverse transcriptase inhibitors; NRTI, nucleoside reverse transcriptase inhibitors; PI, protease inhibitors; INSTIs, integrase strand transfer inhibitors. Total: LLV samples genotypled by iLLV or pLLV; ABC, abacavir; AZT, zidovudine; FTC, emtricitabine; 3TC, lamivudine; TDF, tenofovir; EFV, efavirenz; ETR, etravirine; NVP, nevirapine; RPV, rilpivirine; BIC, bictegravir; CAB, cabotegravir; DTG, dolutegravir; EVG, elvitegravir; RAL, raltegravir.

Analysis revealed that 26.32% (35/133) of the cohort harboured DRMs against at least one drug class (Figure 2A). Utilizing our dual genotyping strategy, we further categorized these DRMs by source: DRMs were detected in plasma RNA in 15.04% (20/133) of participants, indicating actively replicating resistant virus. Solely proviral DNA-derived DRMs (archived resistance) were found in 11.28% (15/133). Resistance to NNRTIs was the most common, detected in 20.30% (27/133) of patients. Plasma RNA sequencing contributed DRMs in 12.78% (17/133) of cases for this class, whereas proviral DNA alone revealed NNRTI DRMs in 7.52% (10/133). Major NNRTI resistance mutations were identified in 9.02% (12/133) of the cohort, primarily driven by G190A/S/E (4.51%) and K103N (3.01%). In contrast, accessory/polymorphic mutations were more frequent. Notably, V179D/E was the most prevalent accessory mutation (9.02%), often detected alongside other DRMs (Figure 2C). Resistance to nucleoside reverse transcriptase inhibitors (NRTIs) was observed in 12.03% (16/133) of cases, with 6.77% detected via RNA and 5.26% via DNA, largely attributable to the M184V/I mutation (9.77%, 13/133); the K65R mutation was also present in 5.26% (7/133) of individuals (Figure 2B). Notably, mutations such as L74V, Y115F, and K70E were detected exclusively in proviral DNA, characteristic of archived NRTI resistance. Integrase strand transfer inhibitor (INSTI) resistance was less frequent, identified in 3.76% (5/133) of participants. Intriguingly, these mutations were detected through different pathways, offering insights into their origins. Among the five cases, DRMs (G163R/K and A128T/E157Q) in three patients with prior INSTI exposure were detected, suggesting possible archived resistance or low-level persistence from past selective pressure. In contrast, DRMs (A128T/E157Q and S230R) were found in two patients without documented INSTI exposure, which may represent transmitted drug resistance or natural polymorphisms.

### DRM distribution across HIV-1 subtypes

The prevalence of DRMs across the major HIV-1 subtypes was as follows: subtype B (100%, 1/1), CRF08_BC (33.33%, 3/9), CRF01_AE (29.41%, 5/17), subtype C (25.00%, 1/4), and CRF07_BC (24.51%, 25/102). It is important to note that a chi-square test found no statistically significant association between viral subtype and the likelihood of harbouring DRMs (Table 3).

### Drug-specific resistance levels

**Figure 3:**
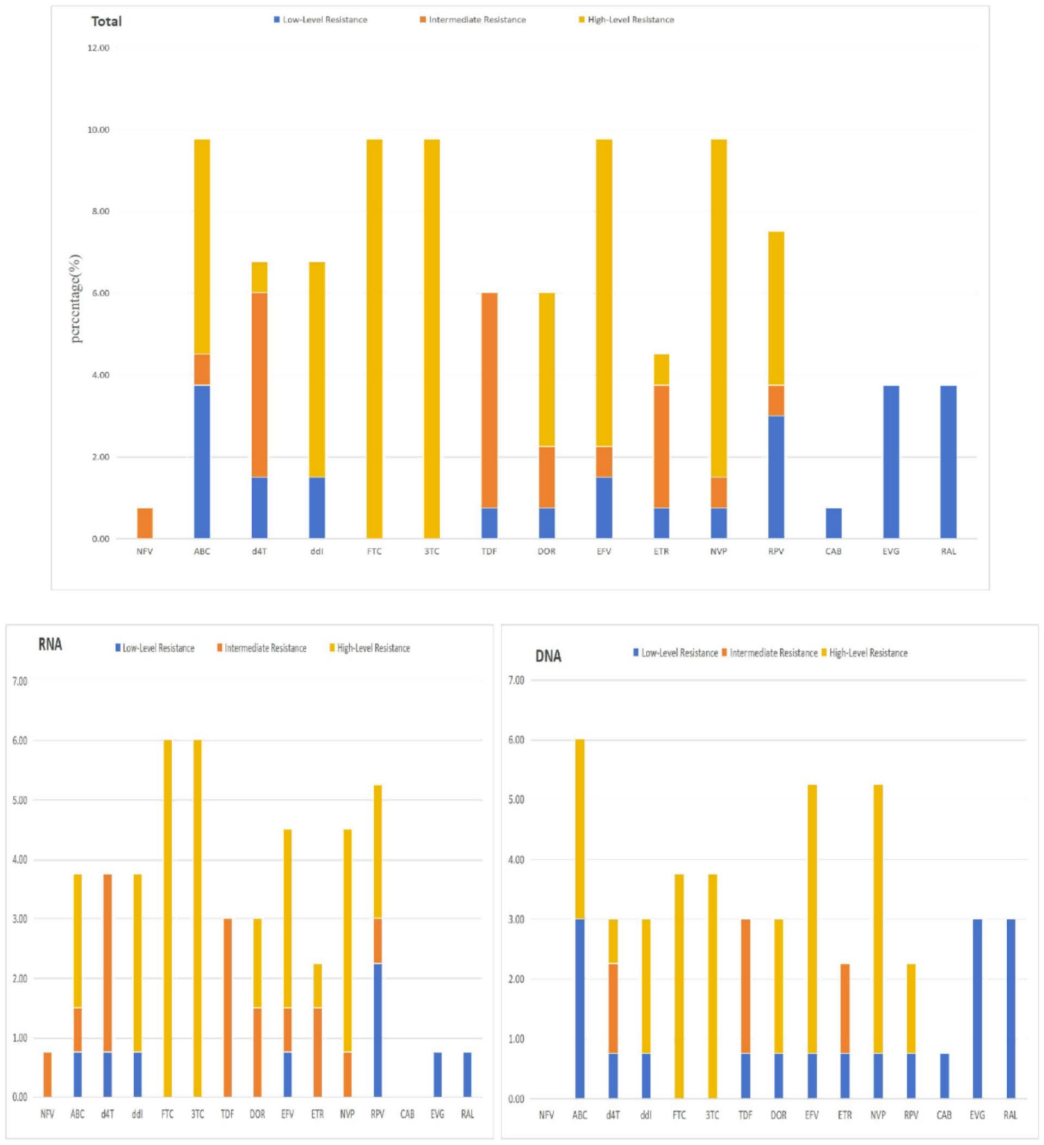
Predicted resistance to antiretroviral drugs among HIV-1 *pol* sequences with DRMs in PLWH with LLV in Chongqing. Different drug resistance levels of four classes of antiretroviral drugs predicted by the Stanford HIV Drug Resistance Database in LLV samples.

The highest rate of high-level resistance was observed for lamivudine (3TC) and its analogue emtricitabine (FTC), both at 9.77% (13/133). Tenofovir disoproxil fumarate (TDF) and stavudine (d4T) were associated with the highest proportions of intermediate-level resistance, at 5.26 and 4.51%, respectively. In contrast, abacavir (ABC) and rilpivirine (RPV) were most frequently linked to low-level resistance, with rates of 3.76 and 3.01%. An assessment of DRM prevalence against key agents in China’s national free ART program revealed that nevirapine (NVP), ABC, 3TC, and efavirenz (EFV) each had a DRM prevalence of 9.77% (13/133), while TDF showed a prevalence of 6.02% (8/133). A comprehensive visualization of these resistance profiles is provided in Figure 3.

### Risk factors for drug resistance

Univariate logistic regression identified initiation of ART within 1 year of diagnosis as a factor associated with significantly reduced odds of developing DRMs (OR = 0.302, 95% CI: 0.101–0.903, *p* = 0.032). However, this association was not sustained in the final multivariable model after adjusting for potential confounders and stratifying by LLV type (Table 2). In the adjusted analysis, a baseline CD4^+^ T-cell count below 100 cells/μL emerged as a strong, independent risk factor for DRMs, but this was confined specifically to the iLLV subgroup [adjusted OR (aOR) = 8.333, *p* = 0.006]. No other demographic or clinical variables were independently associated with DRMs in the final model (Table 4). To further explore the potential influence of genotyping source, we compared baseline characteristics between patients with and without DRMs, stratified by RNA-based and DNA-based genotyping (Table 5). The comparisons revealed generally consistent baseline characteristics across both genotyping groups, with only a marginal difference observed in baseline CD4^+^ T-cell count within the DNA group (*p* = 0.053).

**Table 4:** Multivariate logistic regression analysis of factors associated with drug resistance mutations (DRMs) in patients with persistent low-level viremia (pLLV) and isolated low-level viremia (iLLV).

Variables	pLLV						iLLV					
	Total		DRMs		OR	*p*-value	Total		DRMs		OR	*p*-value
Sex, *n* (%)	65	100.00%	20	30.77%			68	100.00%	15	22.06%		
Male	47	72.31%	15	31.91%	1.219	0.747	52	76.47%	11	21.15%	0.805	0.746
Female	18	27.69%	5	27.78%	1		16	23.53%	4	25.00%	1	
Age, *n* (%)												
<30	5	7.69%	2	40.00%	1.600	0.656	9	13.24%	0	0.00%	0.000	0.999
30–39	14	21.54%	7	50.00%	2.400	0.246	15	22.06%	3	20.00%	0.607	0.526
40–49	12	18.46%	2	16.67%	0.480	0.435	10	14.71%	3	30.00%	1.041	0.961
50–59	17	26.15%	4	23.53%	0.738	0.698	10	14.71%	2	20.00%	0.607	0.583
≥60	17	26.15%	5	29.41%	1		24	35.29%	7	29.17%	1	
Transmission category, *n* (%)												
MSM	12	18.46%	5	41.67%	1		13	19.12%	0	0.00%	1	
HSX	52	80.00%	15	28.85%	0.568	0.391	53	77.94%	15	28.30%	637687404.996	0.999
Other	1	1.54%	0	0.00%	0.000	1.000	2	2.94%	0	0.00%	1.000	1.000
WHO classification stage, *n* (%)												
1	21	32.31%	6	28.57%	1		45	66.18%	11	24.44%	1	
2	2	3.08%	1	50.00%	2.500	0.540	0	0.00%	0			
3	11	16.92%	4	36.36%	1.429	0.652	8	11.76%	1	12.50%	0.442	0.467
4	31	47.69%	9	29.03%	1.023	0.971	15	22.06%	3	20.00%	0.773	0.725
Baseline CD4^+^ T-cell count, *n* (%)												
<100	26	40.00%	7	26.92%	0.798	0.734	18	26.47%	9	50.00%	8.333	0.006
100–250	20	30.77%	7	35.00%	1.167	0.821	22	32.35%	3	13.64%	1.316	0.753
≥250	19	29.23%	6	31.58%	1		28	41.18%	3	10.71%	1	
Baseline viral load, *n* (%)												
<10,000	15	23.08%	7	46.67%	1		31	45.59%	10	32.26%	1	
10,000–100,000	11	16.92%	2	18.18%	0.254	0.144	11	16.18%	1	9.09%	0.210	0.162
100,000–1,000,000	25	38.46%	5	20.00%	0.286	0.082	21	30.88%	4	19.05%	0.494	0.297
≥1,000,000	14	21.54%	6	42.86%	0.857	0.837	5	7.35%	0	0.00%	0.000	0.999
CD4 count during low-level viremia (LLV), *n* (%)												
<200	20	30.77%	4	20.00%	0.300	0.087	20	29.41%	7	35.00%	2.288	0.255
200–450	23	35.38%	6	26.09%	0.424	0.179	27	39.71%	4	14.81%	0.739	0.697
≥450	22	33.85%	10	45.45%	1		21	30.88%	4	19.05%	1	
VL During LLV, copies/mL												
<150	23	35.38%	6	26.09%	1		26	38.24%	4	15.38%	1	
150–300	23	35.38%	7	30.43%	1.240	0.744	17	25.00%	2	11.76%	0.733	0.738
≥300	19	29.23%	7	36.84%	1.653	0.455	25	36.76%	9	36.00%	3.094	0.099
Time from diagnosis to initiation of ART												
Unknown	21	32.31%	8	38.10%	1		34	50.00%	10	29.41%	1	
<1	20	30.77%	3	15.00%	0.287	0.105	19	27.94%	2	10.53%	0.282	0.131
1–2	13	20.00%	5	38.46%	1.016	0.983	6	8.82%	1	16.67%	0.480	0.526
≥2	11	16.92%	4	36.36%	0.929	0.923	9	13.24%	2	22.22%	0.686	0.670
Initial treatment regimen												
2NRTIs + NNRTIs	32	49.23%	11	34.38%	1		49	72.06%	14	28.57%	1	
2NRTIs + PIs	3	4.62%	0	0.00%	0.000	0.999	3	4.41%	0	0.00%	0.000	0.999
2NRTIs + INSTIs	5	7.69%	1	20.00%	0.477	0.530	3	4.41%	0	0.00%	0.000	0.999
NRTIs + INSTIs	21	32.31%	8	38.10%	1.175	0.782	11	16.18%	1	9.09%	0.250	0.206
Other	4	6.15%	0	0.00%	0.000	0.999	2	2.94%	0	0.00%	0.000	0.999

pLLV, persistent low-level viremia; iLLV, isolated low-level viremia; aOR, adjusted odds ratio; CI, confidence interval; MSM, men who have sex with men; HSX, heterosexual contact; VL, viral load; NRTI, nucleoside reverse transcriptase inhibitor; NNRTI, non-nucleoside reverse transcriptase inhibitor; INSTI, integrase strand transfer inhibitor. Statistical model: Multivariate logistic regression analysis was performed separately for the pLLV and iLLV subgroups. Reference categories: For each categorical variable, the reference group is the category against which others are compared (e.g., Female for Sex, ≥60 for Age, MSM for Transmission route, ≥250 for Baseline CD4^+^, <10,000 for Baseline VL, <150 for VL during LLV, and 2NRTIs + NNRTIs for Initial ART regimen). Excluded variables: Marital Status, Occupation, and Education Level: These sociodemographic variables were not included in the final multivariate model for either subgroup. They showed no significant association (*p* > 0.2) in preliminary stratified univariate analyses and were omitted to maintain model parsimony and table clarity.

**Table 5:** Comparison of demographic and clinical characteristics by drug resistance status, stratified by genotyping source (RNA vs. DNA), among PLWH with low-level viremia.

Variables	RNA								DNA							
	All (*N* = 95)		DRMs (*N* = 20)		Non-DR (*N* = 75)		*χ* ^2^	*p*	All (*N* = 38)	DRMs (*N* = 15)		Non-DR (*N* = 23)			*χ* ^2^	*p*
Sex, *n* (%)	95	100.00%	20	21.05%	75	78.95%	0.023	0.880	38	100.00%	15	39.47%	23	60.53%	0.122	0.727
Male	70	73.68%	15	21.43%	55	78.57%			29	76.32%	11	37.93%	18	62.07%		
Female	25	26.32%	5	20.00%	20	80.00%			9	23.68%	4	44.44%	5	55.56%		
Age, *n* (%)																
<30	10	10.53%	1	10.00%	9	90.00%	3.025	0.554	4	10.53%	1	25.00%	3	75.00%	4.387	0.356
30–39	24	25.26%	7	29.17%	17	70.83%			5	13.16%	3	60.00%	2	40.00%		
40–49	11	11.58%	3	27.27%	8	72.73%			11	28.95%	2	18.18%	9	81.82%		
50–59	18	18.95%	2	11.11%	16	88.89%			9	23.68%	4	44.44%	5	55.56%		
≥60	32	33.68%	7	21.88%	25	78.13%			9	23.68%	5	55.56%	4	44.44%		
Transmission category, *n* (%)																
MSM	20	21.05%	3	15.00%	17	85.00%	1.194	0.551	5	13.16%	2	40.00%	3	60.00%	0.671	0.715
HSX	73	76.84%	17	23.29%	56	76.71%			32	84.21%	13	40.63%	19	59.38%		
Other	2	2.11%	0	0.00%	2	100.00%			1	2.63%	0	0.00%	1	100.00%		
WHO classification stage, *n* (%)																
1	50	52.63%	10	20.00%	40	80.00%	1.498	0.683	16	42.11%	7	43.75%	9	56.25%	0.476	0.788
2	2	2.11%	1	50.00%	1	50.00%			0	0.00%	0		0			
3	15	15.79%	4	26.67%	11	73.33%			4	10.53%	1	25.00%	3	75.00%		
4	28	29.47%	5	17.86%	23	82.14%			18	47.37%	7	38.89%	11	61.11%		
Baseline CD4^+^ T-cell count, *n* (%)																
<100	30	31.58%	7	23.33%	23	76.67%	0.271	0.873	14	36.84%	9	64.29%	5	35.71%	5.891	0.053
100–250	32	33.68%	7	21.88%	25	78.13%			10	26.32%	3	30.00%	7	70.00%		
≥250	33	34.74%	6	18.18%	27	81.82%			14	36.84%	3	21.43%	11	78.57%		
Baseline viral load, *n* (%)																
<10,000	31	32.63%	9	29.03%	22	70.97%	3.71	0.294	15	39.47%	8	53.33%	7	46.67%	2.284	0.516
10,000–100,000	14	14.74%	1	7.14%	13	92.86%			8	21.05%	2	25.00%	6	75.00%		
100,000–1,000,000	36	37.89%	6	16.67%	30	83.33%			10	26.32%	3	30.00%	7	70.00%		
≥1,000,000	14	14.74%	4	28.57%	10	71.43%			5	13.16%	2	40.00%	3	60.00%		
Viral load (LLV), *n* (%)																
<150	41	43.16%	7	17.07%	34	82.93%	0.932	0.627	8	21.05%	3	37.50%	5	62.50%	1.9	0.387
150–300	28	29.47%	6	21.43%	22	78.57%			12	31.58%	3	25.00%	9	75.00%		
≥300	26	27.37%	7	26.92%	19	73.08%			18	47.37%	9	50.00%	9	50.00%		
Initial treatment regimen																
2NRTIs + NNRTIs	62	65.26%	16	25.81%	46	74.19%	4.094	0.393	19	50.00%	9	47.37%	10	52.63%	3.969	0.410
2NRTIs + PIs	3	3.16%	0	0.00%	3	100.00%			3	7.89%	0	0.00%	3	100.00%		
2NRTIs + INSTIs	5	5.26%	0	0.00%	5	100.00%			3	7.89%	1	33.33%	2	66.67%		
NRTIs + INSTIs	21	22.11%	4	19.05%	17	80.95%			11	28.95%	5	45.45%	6	54.55%		
Other	4	4.21%	0	0.00%	4	100.00%			2	5.26%	0	0.00%	2	100.00%		
Treatment regimen during low viral load																
2NRTIs + NNRTIs	38	40.00%	7	18.42%	31	81.58%	5.053	0.282	13	34.21%	4	30.77%	9	69.23%	6.036	0.196
2NRTIs + PIs	9	9.47%	3	33.33%	6	66.67%			4	10.53%	1	25.00%	3	75.00%		
2NRTIs + INSTIs	1	1.05%	0	0.00%	1	100.00%			1	2.63%	0	0.00%	1	100.00%		
NRTIs + INSTIs	46	48.42%	9	19.57%	37	80.43%			17	44.74%	7	41.18%	10	58.82%		
Other	1	1.05%	1	100.00%	0	0.00%			3	7.89%	3	100.00%	0	0.00%		

## Discussion

This investigation provides, to our knowledge, the first comprehensive analysis of the drug resistance and viral genotypic profiles in ART-experienced PLWH presenting with LLV in Chongqing, a major HIV epicenter in Southwest China. Our data reveal a considerable prevalence of drug resistance mutations, with more than a quarter (26.32%) of the cohort carrying DRMs. A striking finding was the predominance of NNRTI resistance, which accounted for 19.55% of all cases and constituted the majority of the detected DRMs. Notably, the combined RNA and proviral DNA genotyping strategy markedly improved the success rate of resistance testing (63.33%) in this low-VL cohort. These findings address a significant knowledge gap and provide critical evidence to guide the clinical management of LLV in resource-limited settings.

In this study, DRMs detected in plasma viral RNA likely represent actively replicating virus and are directly implicated in the current episode of LLV. In contrast, DRMs identified solely in proviral DNA represent archived resistance, integrated into the host genome, which may reflect past selective drug pressure or minority variants not currently dominant in plasma. While archived mutations may not be driving immediate viremia, they constitute a reservoir that can re-emerge and compromise future regimen efficacy, thus holding significant clinical prognostic value. The overall prevalence of LLV in our cohort was 4.25% (210/4,941), which is substantially lower than rates reported in a national Kenyan cohort (18.5%) (15) and a European multicenter cohort (46%) (16). However, it aligns with findings from other Chinese regions, such as Zhengzhou (3.32%) (11) and Guangdong (3.3%) (12). This relatively low LLV rate in Chongqing may reflect particularities of the local ART management system and follow-up frequency. The DRM rate we detected (26.32%) was lower than the high rates reported in South Africa (79.2%) (4), Henan, China (42.3%) (11), and Guangdong, China (47.06%) (12), but higher than rates in some US cohorts (17%) (17). These pronounced geographical disparities likely arise from differences in ART regimen composition and historical use (e.g., extensive NNRTI exposure), overall treatment duration, and resistance detection methodologies.

Our finding that NNRTI resistance was the most prevalent (19.55%) aligns with China’s historical reliance on first-generation NNRTIs (EFV and NVP). However, a nuanced interpretation is warranted. The resistance profile is bimodal: a subset of patients harbored major mutations (e.g., G190A/S/E, K103N) that confer high-level resistance and pose a clear risk to current NNRTI-based regimens. Conversely, a larger proportion exhibited accessory mutations such as V179D/E—the single most common mutation in our cohort (9.02%)—may not necessitate regimen change unless combined with other DRMs. Therefore, while the prevalence of NNRTI mutations is substantial, the clinical burden is primarily concentrated in the ~9% of patients with high-level resistance mutations. This distinction is critical for resource-efficient management: patients with accessory mutations alone may be managed with enhanced adherence counseling and monitoring, whereas those with major mutations likely require regimen optimization. The pattern of NNRTI resistance we observed characterized by this bimodal distribution,finds parallels in other settings. This pattern is consistent with findings from a Zhengzhou study, which also reported NNRTIs as the most compromised drug class (35.58%), with V179D/E among the major mutations (11). It reflects the extensive historical and ongoing use of efavirenz and nevirapine-based regimens under China’s national free ART program. Beyond China, a European cohort documented a 35% DRM rate in LLV, linking newly emergent mutations to subsequent VF (16). A study from Cameroon further underscored the risk, finding that 82.2% of individuals with LLV and a VL ≥ 200 copies/mL harbored DRMs (18). Collectively, these data substantiate the clinical value of resistance testing for PLWH with LLV, especially when the VL is ≥200 copies/mL.

The NRTI resistance rate was 12.03%, prominently featuring the M184V/I mutation (9.77%). This finding directly reflects the sustained selective pressure exerted by the long-term use of 3TC or FTC as backbone agents in ART regimens. The M184V/I mutation confers high-level resistance to 3TC/FTC and is associated with potential cross-resistance, a pattern consistent with global reports and observations from other Chinese cohorts in Guangdong (12) and Zhengzhou (11).

The prevalence of INSTI resistance was low (3.76%, 5/133) in our study population. This finding is consistent with the local treatment history in Chongqing, where INSTI-based regimens have been more recently introduced as first-line options, leading to relatively limited long-term population-level exposure thus far (14). Although the detected INSTI-associated mutations are generally associated with low-level resistance and are rare in treatment-naïve or LLV populations, their clinical relevance in LLV remains unclear. Their detection alone, in the absence of virologic failure or high-level resistance evidence, should not prompt immediate regimen change, particularly for high-barrier INSTIs such as dolutegravir (DTG) and bictegravir (BIC). However, with the escalating global and national adoption of INSTI-based regimens, proactive and ongoing monitoring of INSTI resistance is imperative. This caution is supported by reports from Taiwan (19) and Zhengzhou (11), which highlight the potential for INSTI resistance emergence in the context of LLV.

Our findings underscore the practical challenge of conducting resistance surveillance in the LLV setting, the utility of conventional plasma RNA genotyping for resistance surveillance is limited by its sensitivity, which is generally insufficient to reliably amplify viral RNA at the low VL characteristic of LLV (typically <1,000 copies/mL). The combined RNA/DNA genotyping strategy proved to be a robust method for this low-VL cohort, enabling successful pol and IN gene sequencing in 63.33% (133/210) of the identified LLV patients. This approach was essential, as proviral DNA genotyping supplemented resistance data in nearly 30% of cases where RNA-based testing was not feasible, thereby preventing false negatives and enabling the detection of “archived” DRMs (6, 20)-historical resistance variants integrated into the host genome that can inform the risk of future treatment failure. The efficacy of proviral DNA genotyping in LLV populations has been corroborated by a study in Botswana (21), reinforcing its value for resource-limited settings. Our data suggest that proviral DNA testing should be prioritized when RNA genotyping is not feasible, particularly for individuals with VLs in the 50–200 copies/mL range. Resistance mutations found only in proviral DNA likely represent archived resistance, reflecting either past drug selection or transmitted resistant virus at infection. Although not directly driving the current LLV, they form a latent resistant reservoir that could be reactivated during future regimen changes, posing a risk to treatment efficacy. Therefore, detecting archived resistance is important for informing long-term treatment strategies, especially when reusing drug classes from a patient’s history.

The occurrence of LLV is multifactorial, potentially involving ongoing low-level viral replication from reservoirs, drug selection pressure, incomplete immune control, and intermittent adherence. Our RNA/DNA genotyping strategy helps differentiate between drug resistance selected by current replication and the persistence of a historical resistance reservoir. Future longitudinal studies are needed to elucidate how these resistance mutations evolve amidst the interplay between viral reservoir dynamics and immune surveillance.

In exploring risk factors for DRMs, univariate analysis suggested a protective effect of initiating ART within 1 year of diagnosis (OR = 0.302, *p* = 0.032). However, this association was not sustained in the multivariable model after adjusting for confounders and stratifying by LLV type. Conversely, a baseline CD4^+^ T-cell count <100 cells/μL was significantly associated with DRMs, but this association was confined to the iLLV subgroup (aOR = 8.333, *p* = 0.006). This finding suggests that advanced immunosuppression at ART initiation, which may reflect prolonged untreated infection or delayed treatment initiation, is linked to resistance in individuals experiencing transient viremic episodes. The biological basis for this association being specific to iLLV remains unclear but warrants further investigation. However, it is crucial to acknowledge that suboptimal adherence is a well-established driver of both LLV and the emergence of DRMs. The absence of objective adherence data (e.g., pill counts, electronic monitoring, or therapeutic drug monitoring) in our study represents a major limitation, as it constrains our ability to distinguish between true virologic failure due to resistance versus non-adherence. The observed association between low baseline CD4^+^ count and DRMs in iLLV patients, while statistically significant, may be partially confounded by unmeasured adherence patterns. It should be noted, however, that this subgroup analysis had a limited sample size, and this association requires further confirmation in larger studies. A recent systematic review of 7,508 LLV patients further confirmed a pooled DRM prevalence of 28.74%, and the presence of DRMs was a significant barrier to subsequent virologic suppression (OR = 0.29) (6). Additionally, Lan et al. demonstrated that ART duration exceeding 1 year was an independent predictor of DRM risk (12). These findings collectively underscore the critical importance of initiating antiretroviral therapy early in preventing the emergence of drug resistance. A low baseline CD4 count reflects advanced immunosuppression and is often associated with prolonged untreated infection and a higher risk of pre-existing resistance (22). Among iLLV patients, this subgroup exhibits a high risk of DRMs, warranting targeted clinical management. Li et al. (23) reported suboptimal adherence in 38.05% of LLV patients, while enhanced adherence counseling significantly improved virologic outcomes. Therefore, for iLLV patients with low baseline CD4 counts, we recommend proactive adherence assessment combined with integrated RNA/DNA genotyping for resistance testing. Where indicated, optimization or switching to high-barrier regimens (e.g., INSTI-based) should be considered to prevent the emergence of resistance and avert virologic failure.

Study Limitations and Future Directions: (1) The cross-sectional design precludes causal inference; (2) The sample size (*n* = 133) precluded adequate analysis of rare subtypes or mutations; (3) The lack of therapeutic drug monitoring and objective adherence data is a major limitation. This absence likely confounds our estimates of DRM prevalence and complicates the interpretation of risk factor analyses, as we cannot adjust for or stratify by adherence levels. (4) Furthermore, the subgroup analyses (e.g., stratified by LLV type) may have been underpowered due to limited sample sizes, which warrants validation in larger cohorts. Subsequent work will involve enhanced follow-up of these cases to observe outcomes. Future studies should: (1) Employ prospective cohort designs, incorporating therapeutic drug monitoring (TDM) and precise adherence assessment tools (e.g., electronic pillboxes), combined with longitudinal VL and resistance monitoring, to elucidate the causal relationship between resistance evolution and VF; (2) Explore the application of Next-Generation Sequencing (NGS) (24) to improve the detection of low-abundance DRMs; (3) Expand the research scope and cohort size, integrating data on viral subtypes, epidemic history, and treatment practices from more regions in Southwest China to comprehensively validate regional resistance patterns and develop targeted management strategies.

## Conclusion

This study systematically characterizes a substantial burden of drug resistance mutations (26.32% overall), predominantly against NNRTIs (19.55%), among ART-experienced PLWH with LLV in Chongqing. Critically, by employing a combined plasma RNA and proviral DNA genotyping strategy, we successfully differentiated between actively replicating resistance (plasma RNA-derived) and archived resistance (proviral DNA-derived), both contributing to the overall resistance pool. a region dominated by the CRF07_BC subtype. While initiation of ART within 1 year of diagnosis was associated with a reduced prevalence of DRMs in univariate analysis, but this was not confirmed in multivariable analysis. In contrast, a baseline CD4^+^ T-cell count <100 cells/μL was independently associated with DRMs in patients with iLLV. The findings highlight that comprehensive resistance surveillance in LLV must capture archived resistance, which informs long-term therapeutic vulnerability, even if not directly causative of the current low-level viremia. The integrated RNA and proviral DNA genotyping approach proved essential for successful resistance surveillance in this low viral load cohort.

### Clinical implications and recommendations

Based on our findings, we propose the following clinical management recommendations: For patients with LLV in the 200–1,000 copies/mL range, plasma RNA-based genotypic resistance testing should be attempted first. If the VL is persistently below 200 copies/mL or RNA testing fails, proviral DNA genotyping serves as a valuable supplementary tool to uncover archived resistance. Patients with isolated LLV and a baseline CD4^+^ T-cell count <100 cells/μL should be considered at high risk for resistance; proactive genotypic testing (combined RNA/DNA) and enhanced adherence counseling are advised. Furthermore, in interpreting results, a distinction should be made between major NNRTI resistance mutations and polymorphic/accessory mutations (e.g., V179D/E); clinical decisions regarding regimen switching should rely primarily on major mutations.

## Data Availability

The datasets presented in this study can be found in online repositories. The names of the repository/repositories and accession number(s) can be found in the article/supplementary material.

## Footnotes

1
https://hivdb.stanford.edu/hivdb/by-sequences/

2
http://dbpartners.stanford.edu:8080/RegaSubtyping/stanford-hiv/typingtool/

3
https://www.hiv.lanl.gov/
